# The cognitive and motor effects of immersive virtual reality in individuals with neurocognitive disorder: randomized controlled trial protocol

**DOI:** 10.1016/j.clinsp.2025.100704

**Published:** 2025-07-03

**Authors:** Maristela Chaya, Jessica Maria Ribeiro Bacha, Paula Lagos, Vitória Terzian, Ana Claudia Souza, Luciana dos Anjos, Julia Maria D'Andrea Greve, Regina Mikisian Magaldi, Gislaine Gil, Alexandre Leopold Busse, José Eduardo Pompeu

**Affiliations:** a Faculdade de Medicina, Universidade de São Paulo (FMUSP), São Paulo, SP, Brazil; b Faculdade Sírio-Libanês, São Paulo, SP, Brazil

**Keywords:** Cognition, Older adults, Aged, Virtual reality exposure therapy, Physiotherapy

## Abstract

**Objective:**

To assess the cognitive and motor effects of an intervention utilizing commercial immersive virtual reality (IVR) games in older adults diagnosed with mild neurocognitive disorder (mild NCD) or mild major neurocognitive disorder and compare these effects with those of a motor-cognitive integrated exercise program.

**Methods:**

This randomized controlled trial will include volunteers aged 60 years and older diagnosed with mild NCD or mild major NCD. Participants will be randomly assigned to two groups, each undergoing two 45-minute sessions weekly for seven weeks. The Virtual Reality Group (VRG) will engage in six IVR games, while the Exercise Group (EG) will perform integrated motor-cognitive exercises. Outcomes will be measured using the mini-BESTest, Dynamic Gait Index, Box and Block Test, 1-minute sit-to-stand test, Grip Strength Test, Neurocognitive Battery, Word Accentuation Test, Patient Health Questionnaire-9, Generalized Anxiety Disorder-7, Montreal Cognitive Assessment, and Functional Activities Questionnaire. Sample size calculation indicates 32 participants (16 per group) to achieve 80 % power with α = 0.05, accounting for 20 % attrition. The trial is registered at the Brazilian Clinical Trials Registry (RBR-2kk9vnh).

**Results:**

It is hypothesized that participants in the VRG will demonstrate greater improvements in cognitive and motor performance compared to the EG.

**Conclusions:**

This study aims to determine whether commercial IVR games can serve as effective cognitive and motor interventions for individuals with mild NCD or mild major NCD.

## Highlights

• IVR games can enhance cognitive and motor training in mild neurocognitive disorders.
• IVR games support cognitive and motor function non-pharmacologically.
• Combining fun and rehab to boost motivation and tech inclusion in older adults.
• IVR games offer an affordable and accessible option for middle-income countries.
• IVR Games may help delay Alzheimer’s in mild neurocognitive disorder.

## Introduction

The older population, which has grown drastically since the beginning of the 21st century, is at a considerably higher risk of brain-aging-related issues.^1^ As the proportion of older individuals increases within the general population, the number of neurocognitive disorders in gerontological practice also rises. Among other challenges, this demands effective interventions for prevention and gerontological rehabilitation.^1^^,^^2^

Mild Neurocognitive Disorder (NCD), also known as mild cognitive impairment, is defined as cognitive decline beyond what would be expected for age and education in one or more cognitive domains (complex attention, executive functions, learning and memory, language, perceptual-motor, or social cognition) based on concerns of the individual, caregiver, or clinician. Yet, these changes do not interfere with the ability to remain independent in daily activities.^3^ It is common in older populations, and its prevalence increases with age.^4^ It is estimated that the global prevalence of mild NCD is between 15 % and 20 % among people aged 60 and older.^5^

Approximately 80 % of patients with mild NCD will have converted to Alzheimer's Disease (AD) after approximately six years of follow-up.^6^ Therefore, the predicted risk of this conversion and the early identification of high-risk mild NCD participants are urgently needed. Since AD is irreversible and faces significant treatment challenges, the key to preventing and treating AD is to take early preventive and intervention strategies. The mild NCD stage provides a “window of opportunity” for AD prevention and treatment.^7^^,^^8^

Based on neuroplasticity, cognitive interventions can provoke brain changes and generate new functional connections.^9^ Thus, to slow the progress of neurodegenerative dementia, cognitive training has been suggested as an approach to improve impaired brain functions.^10^^,^^11^

Another non-pharmacological intervention, in addition to cognitive training, that can help improve mood and preserve cognitive functions is physical exercise,^12^ which can enhance brain metabolism and brain-derived neurotrophic factor, supporting brain plasticity and hippocampal angiogenesis.^13^ Multidomain or multimodal interventions, consisting of two or more interventions, may have even more significant benefits than cognitive training or exercise alone.^14^

A new and promising tool to improve cognitive functioning is Virtual Reality (VR) .^2^^,^^15^ VR is defined by Lange and Pompeu as follows: a three-dimensional environment presented on a screen with which the user can interact using movements or gestures tracked by sensors, gloves, tracking devices, or touchscreens.^16^ VR technology uses human senses (vision, touch, movement) in a virtually created environment. Its advantage is the ability to simulate real-life experiences in a pre-created virtual environment and provide short-term feedback^17^ according to the individual's performance.^18^

VR allows users to experience and interact with computer-generated environments and, in some games, react as they would in real life when performing predetermined tasks.^19^ This method can be used to engage in fun and exciting tasks, thus increasing user motivation.^20^ VR health applications have been evaluated in various studies to address health-related issues in older adults.^21^

Immersive technologies allow users to isolate themselves from the physical world, explore the virtual environment, and create mental representations of the virtual environment, leading to the experience of spatial presence.^22^ Neurological evidence further indicates that the experiences of spatial presence induced by immersive media would increase activity in brain regions associated with cognitive functions.^1^^,^^23^

VR systems within the rehabilitation context can be grouped into systems customized for rehabilitation and commercial systems aimed at a broader entertainment market. The advantage of customized systems developed for rehabilitation is that they follow rehabilitation principles and, therefore, can be intrinsically valuable. On the other hand, commercial systems may be more cost-effective, entertaining, and of higher product quality, but in turn, they require adaptation to find their place as a rehabilitation tool.^24^

The Head Mounted Display (HMD) represents the most immersive VR technology. The literature on HMD-based VR for cognitive rehabilitation is limited. Rehabilitation approaches tested with HMD VR include customized software.^25^ Combining commercial hardware and software enables a more economically sustainable approach by avoiding the development of customized systems, which often entail high costs. This approach not only reduces operational costs in healthcare institutions but also has the potential to slow the progression of cognitive and motor deficits, generating economic benefits by reducing the need for advanced care in the future. This strategic integration can serve as a foundation for public health policies aimed at active aging and the technological inclusion of older populations.

## Objective

To analyze the acceptability and effects of immersive VR combining commercial hardware and software in individuals with mild NCD or mild major NCD and compare it with a motor-cognitive integrated intervention.

### Hypotheses

1. VR interventions will significantly improve postural control, gait, functionality, cognition, and mood more than the motor-cognitive integrated intervention.
2. The authors hypothesize that participants undergoing VR interventions will improve their performance in the games.
3. The authors expect that commercial VR games will be acceptable to older adults with mild NCD or mild major NCD. Although this population may not be familiar with this technology, commercial games were developed for entertainment in an attractive and motivating virtual environment and were easy to use.

### Study design

The study will be a randomized, controlled, and blinded clinical trial. Participants will be divided into two groups: the VR group (VRG) and the exercise group (EG). The study assessments will be conducted before and after the intervention.

## Methods

### Study location

All participants will be recruited from the Older People Memory Clinic of the Clinical Hospital, School of Medicine, University of São Paulo, Brazil.

### Eligibility criteria

#### Inclusion criteria

The following will be included: 1) Older people individuals (> 60-years-old); 2) Both genders; 3) Adequate proficiency in the Portuguese language; and classified in one of the following two categories: (3) Mild NCD – at least one domain with a composite score less than or equal to −1, but without criteria for dementia; (4) Mild Major NCD with a CDR (Clinical Dementia Rating scale) <2; and (5) Agreeing to participate in the study by signing the consent form.

#### Non-inclusion criteria

The following older people individuals will not be included; 1) Presence of delirium, psychotic mental disorders, or substance-related mental disorders, according to DSM-5 criteria; 2) Severe retinal problems or severe visual deficits even with corrective lenses; 3) Uncorrected severe hearing loss; 4) Those with decompensated cardiovascular diseases, such as angina or heart failure; 5) Epilepsy; 6) Motion sickness; 7) Any health issue that prevents them from using VR; 8) Severe clinical conditions with uncontrolled symptoms or instability.

#### Exclusion criteria

Participants who experience cybersickness during the familiarization session and those who miss the first two consecutive sessions will be excluded. Additionally, participants must complete 100 % of the training sessions to remain in the study. They may miss up to 20 % of the sessions, provided that all missed sessions are made up through compensatory sessions, to be scheduled in agreement with the research team.

### Assessments

A trained researcher will provide the participant with an informed consent form. The same researcher, blind to the treatment allocation, will assess all participants at two-time points: before and after the interventions. Participants will be asked not to inform the evaluators about the type of intervention they received.

### Interventions

Participants will be randomly assigned to the VRG or EG groups, with an allocation ratio of 1:1. Participants in the VRG will participate in 14 IVR training sessions lasting 45 min, twice a week. The Oculus Quest 2 Advanced 128Gb VR Headset will be used, attached, and adjusted to the participant's head. A BOBOVR M2 Pro adapter will be used for greater comfort, a support that offers greater comfort, and a magnetic battery with quick replacement and charging and two wireless handheld controllers. In each training session, participants will play three games on odd days and another three games on even days. A trained physiotherapist will accompany all sessions to correct the participants' movements and posture through manual guidance and verbal cues. The level of difficulty progression will be according to the participant's performance. Scores will be recorded for learning analyses. The time required to change the game will be approximately three minutes, and participants will sit in a chair during this period. Individuals in the EG will participate in 14 training sessions of a motor-cognitive integrated intervention, lasting 45 min and having a frequency of twice a week. A trained physiotherapist will conduct the sessions.

### Selection and description of the games

The games require the participant to remain standing and were selected based on their motor and cognitive demands. Table 1 describes the games.

**Table 1:** Brief description of the games. Figures resulting from the game's print screen.

### Selection and description of motor-cognitive integrated intervention

Table 2 describes the exercises of the motor-cognitive integrated intervention.

**Table 2:** Brief description of the exercises.

Description of Multimodal Exercises with integrated cognitive interventions	
Individually supervised interventions by a trained physical therapist. Twice a week, for 45-minutes, over 7-weeks. First day: Exercises 1, 2, 4, 6, 8, and 10. Second day: Exercises 1, 3, 5, 7, 9, and 10.	
**Description**	**Materials**
1. Walking while the participant verbalizes words with the same pre-determined phonetic initial.	None
2. During the plantar flexion movement, the individual must touch colored markers positioned on the wall in a random order pre-defined by the researcher.	Colored EVA circles
3. Execution of a circuit consisting of activities using proprioceptive discs and unstable surfaces, combined with simple mathematical calculations.	Proprioceptive disc and 6 mats (55 × 95 × 3 cm)
4. Performing ball-throwing exercises while the participant verbalizes words belonging to specific semantic categories.	Inflatable ball (20 cm)
5. Performing exercises on a step platform with load, alternating legs according to pre-defined instructions.	Step platform (60 × 28 × 10 cm)
6.Memorizing the stacking order of cones on a chair and replicating this order on another chair positioned 3 m away.	6 colored cones and 2 chairs
7. Maintaining static balance on an unstable surface while the participant claps upon hearing a specific letter of the alphabet announced by the researcher.	3 mats (55 × 95 × 3 cm)
8. Hip adduction exercise with a ball between the knees and hip abduction against elastic resistance around the knees, while the participant recalls and describes figures from a previously presented image.	Inflatable ball (20 cm), elastic band, and printed images
9. Walking with arms extended while holding a ball, performing upper limb abduction movements according to opposite verbal commands.	Inflatable ball (20 cm)
10. Stretching.	None

**Fig. 1:**
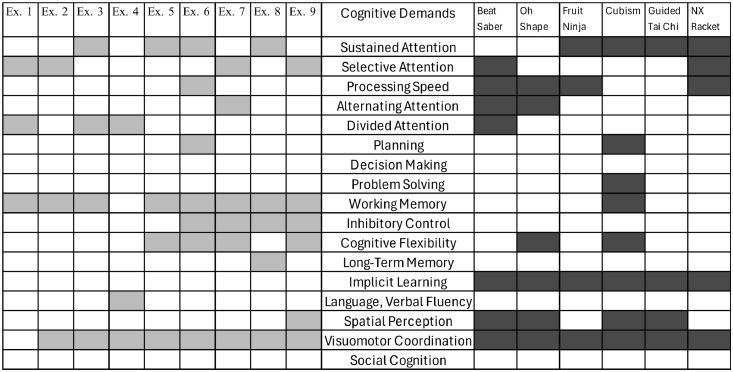
Cognitive domains are stimulated during exercises and games.

**Fig. 2:**
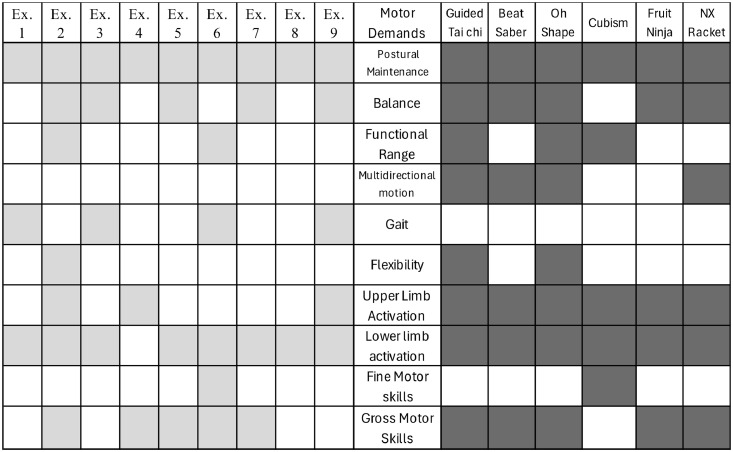
Motor skills stimulated during exercises and games.

Physiotherapists and neuropsychologists mutually agreed to select the exercises and games according to the specific demands. The cognitive and motor demands of the games and exercises were then paralleled, illustrated in Fig. 1, Fig. 2.

### Outcome measures of acceptability

Feasibility will be assessed by the participant's game performance, measured by the scores achieved. Increasing scores indicate that the participant can play and improve their performance in the games. It is the primary measure of the game that promotes motivation.^26^ Acceptability will be evaluated through a game satisfaction questionnaire. The questionnaire consists of nine questions, including participants' perceptions of the games (“What did you think of the game?”; “Would you recommend this game?”; “Would you play this game at home?").

### Clinical outcome measures

Table 3 shows the assessments used as Clinical Outcome Measures.

**Table 3:** Clinical outcome measures and assessments.

Clinical outcome	Assessment
Postural control	Mini-BESTest^27^
Gait	Dynamic Gait Index^28^
Cognitive functions	Neurocognitive Battery of SGHC-FMUSP; Word Accentuation Test^29^; MoCA (Montreal Cognitive Assessment)^30^
Functionality	Functional Activities Questionnaire^31^
Mood	Generalized Anxiety Disorder 7-item^32^; Patient Health Questionnaire-9^33^
Manual dexterity	Box and Blocks^34^
Strength	Hand Grip Test^35^

### Participant timeline

Table 4 illustrates the study participant enrollment process, intervention, and timing of assessments.

**Table 4:** Activity timeline.

	Day 1	Day 2	Days 3‒16	Day 17
Eligibility screening	x			
Initial neuropsychological assessments	x			
Informed consent	x			
Initial physical assessments		x		
Allocation Interventions		x		
			x	
Final neuropsychological assessments				x
Final physical assessments				x

### Sample size

The sample size calculation was based on a review with meta-analysis regarding the effectiveness of VR for individuals with mild cognitive impairment or dementia, which produced small to medium effect sizes using a random effects model (effect size = 0.29) from a total of 11 studies.^36^ Assuming a dropout rate of 20 %, a total sample size of 32 participants (16 per treatment group) would provide a power of 0.8 with a two-tailed α error of 0.05.

### Recruitment

Potentially eligible participants will be identified by the clinical care team at the Older People Memory Outpatient Clinic of the Clinical Hospital, Faculty of Medicine, University of São Paulo. This team will conduct cognitive and mood assessments. If the participant is willing to participate, a qualified person will provide verbal and written information about the study.

### Randomization

Participants will be randomly assigned to the VRG and EG with a 1:1 allocation ratio. A researcher not involved in the study will prepare the randomization schedule from a computer-generated list of random numbers.

### Blinding

It will not be possible to blind the participants from the test and the intervention facilitator. All outcomes will be assessed by a researcher who is blinded to group allocation. Participants will be asked not to disclose their allocation to the physiotherapist conducting the assessments. This team will conduct cognitive and mood assessments. If the participant is willing to participate, a qualified person will provide verbal and written information about the study.

### Data collection

Demographic data will be obtained through electronic forms, including a question regarding previous COVID-19 infection as a demographic variable to be considered. In real-time, trained professionals will conduct questionnaires and assessments, entering the results directly into the Research Electronic Data Capture (REDCap).

Demographic data will be obtained through electronic forms. In real-time, trained professionals will conduct questionnaires and assessments and will enter the results directly into the Research Electronic Data Capture (REDCap).

### Data management and monitoring

All research data will be collected directly in REDCap, at the FM-USP instance, and stored on a server in the USP cloud (MySQL), with automatic internal backups and daily backups on the institutional Google Drive, in addition to firewall protection.

### Data analysis

Continuous variables will be presented as mean and standard deviation, while categorical variables will be expressed as absolute frequencies (n) and percentages ( %). The distribution of the data will be assessed using the Shapiro-Wilk test. The Z-score will be used to normalize non-parametric data. Continuous data will be analyzed using the General Linear Model (GLM) for repeated measures and univariate GLM for group comparisons. The effect size and 95 % Confidence Interval will be reported along with their interpretations. The significance level will be set at α ≤ 0.05, and when applicable, clinical details related to the instruments, such as the minimum detectable change and measurement error, will also be included. Data analyses will be conducted using SPSS version 24.0.

### Adverse events

An adverse event is defined as any undesirable medical occurrence in a participant that does not necessarily have a causal relationship with this intervention. The study team will review any adverse events, assess probable causality, and report on a form.

### Audit

The authors will establish a rigorous quality control program. A trained team and the trial coordinator will ensure adherence to the trial protocols. The University of São Paulo will conduct quality assurance checks to ensure the integrity of randomization, study entry procedures, and data collection.

### Protocol amendments

Any modifications to the protocol that may impact the conduct of the study, potential benefits to the participant, or that may affect participant safety ‒ including changes to study objectives, study design, participant population, sample sizes, study procedures, or significant administrative aspects ‒ will require a formal amendment to the protocol. Such amendments will be agreed upon by the Ethics Committee of the Faculty of Medicine, University of São Paulo, Brazil, and the Brazilian Clinical Trials Registry before implementation and reported to health authorities in accordance with local regulations.

### Dissemination policies

The dissemination will aim to inform a wide range of local, national, and international audiences about the results and conclusions. However, it should be remembered as part of this strategy that the current project is a preliminary work intended to inform a subsequent definitive clinical trial. The goal is to publish the present research in journals that cover relevant medical specialties, preferably those that deposit publications in open-access databases to enhance free dissemination. Additionally, the authors aim to present this research at appropriate national and international conferences.

## Discussion

The current study will compare the effects of VR games with a motor-cognitive integrated intervention on cognitive and motor outcomes in NCD participants. The choice of commercial games as an intervention is justified by their accessibility and potential to engage older adults in a playful manner, promoting social interaction and enjoyment. The literature already suggests that games can improve cognitive functions, not only overall cognitive scores but also memory, attention, and processing speed, along with offering motor benefits.^37^ Several studies consider cognitive-motor training to be a beneficial training method leading to greater cognitive and motor improvements and is essential for better understanding which intervention may be more effective and well-accepted.^38^

Acceptability is a crucial factor for the sustainability of any intervention program. Older adults are expected to feel more motivated to participate in activities that are perceived as enjoyable and stimulating. However, it is also essential to consider the barriers that may arise; all health disciplines should challenge age bias and embrace and support the use of technology by older adults, promoting digital inclusion for these individuals.^39^

The expected results of this study may significantly contribute to the formulation of intervention strategies more suited to the needs of older adults, promoting healthy and active aging. Furthermore, comparing interventions will allow for identifying approaches that may be more effective in managing NCD and mild major NCD, providing directions for future clinical practices and public health policies.

## Abbreviations

AD, Alzheimer's Disease; CDR, Clinical Dementia Rating scale; EG, Exercise Group; HMD, Head Mounted Display; IVR, Immersive Virtual Reality; Mini-BESTest, Mini-Balance Evaluation Systems Test; MoCA, Montreal Cognitive Assessment; SGHC-FMUSP, Geriatrics Service of the Clinical Hospital of the Faculty of Medicine of the University of São Paulo; NCD, Neurocognitive disorder; VR, Virtual Reality; VRG, Virtual Reality Group; REDCap, Research Electronic Data Capture.

## Trial status

To date, the authors have recruited approximately 50 % of participants and collections have begun.

Availability of data and materials: Not applicable.

## Consent for publication

Not applicable.

## Ethics approval and consent to participate

This study was approved by the Ethics Committee of the Faculty of Medicine of the University of São Paulo, Brazil (5.959.554), Certificate of Submission for Ethical Assessment: (CAAE: 67,444,823.0.0000.0068); Free and Informed Consent Form (Additional file). This study was registered in the Brazilian Clinical Trials Registry (RBR-2kk9vnh) on October 25, 2023.

## Funding

This research received no specific grant from funding agencies in the public, commercial, or not-for-profit sectors.

## Authors’ contributions

Chaya M, Bacha JMR, Lagos P, Terzian V, Souza AC, dos Anjos L, Gil G, Busse AL, Pompeu JE were responsible for study conceptualization and methodology. Chaya M, Souza AC, Busse AL, Pompeu JE were responsible for validation, and formal analysis. Chaya M, Souza AC, dos Anjos L, Terzian V, Lagos P, Busse AL, Pompeu JE contributed to investigation and original draft preparation. Bacha JMR, Busse AL, Pompeu JE were responsible for manuscript review and editing. Chaya M, Bacha JMR, Greve JMA, Magaldi RM, Gil G, Busse AL, Pompeu JE contributed to data visualization. Chaya M, Bacha JMR, dos Anjos L, Souza AC, Lagos P, Terzian V, Greve JMA, Magaldi RM, Busse AL, Pompeu JE were responsible for resources and data curation. Chaya M, Bacha JMR, Souza AC, Greve JMA, Magaldi RM, Gil G, Busse AL, Pompeu JE were responsible for project administration. Chaya M, Greve JMA, Magaldi RM, Gil G, Busse AL, Pompeu JE were responsible for funding acquisition.

## Declaration of competing interest

The authors declare no conflicts of interest.
